# A Comprehensive Physiologically Based Pharmacokinetic Model for Predicting Vildagliptin Pharmacokinetics: Insights into Dosing in Renal Impairment

**DOI:** 10.3390/ph17070924

**Published:** 2024-07-10

**Authors:** Mahnoor Pasha, Ammara Zamir, Muhammad Fawad Rasool, Hamid Saeed, Tanveer Ahmad, Nawaf Shalih Alqahtani, Lamya Saif Alqahtani, Faleh Alqahtani

**Affiliations:** 1 Department of Pharmacy Practice, Faculty of Pharmacy, Bahauddin Zakariya University, Multan 60800, Pakistan; mahnoorpashaoc15@gmail.com (M.P.); ammarazamir20@gmail.com (A.Z.); 2 Section of Pharmaceutics, University College of Pharmacy, Allama Iqbal Campus, University of the Punjab, Lahore 54000, Pakistan; hamid.pharmacy@pu.edu.pk; 3 Institute for Advanced Biosciences (IAB), CNRS UMR5309, INSERM U1209, Grenoble Alpes University, 38700 La Tronche, France; tanveer.ahmad@univ-grenoble-alpes.fr; 4 King Abdulaziz Medical City, Riyadh Region Ministry of National Guard, Health Affairs, Riyadh 11426, Saudi Arabia; alkahtanina3@mngha.med.sa; 5 Department of Cardiology, Prince Sultan Cardiac Center, Riyadh 11625, Saudi Arabia; lalqahtani@pscc.med.sa; 6 Department of Pharmacology and Toxicology, College of Pharmacy, King Saud University, Riyadh 11451, Saudi Arabia

**Keywords:** vildagliptin, type 2 diabetes, physiologically based pharmacokinetic model, chronic kidney disease

## Abstract

Physiologically based pharmacokinetic (PBPK) modeling is of great importance in the field of medicine. This study aims to construct a PBPK model, which can provide reliable drug pharmacokinetic (PK) predictions in both healthy and chronic kidney disease (CKD) subjects. To do so, firstly a review of the literature was thoroughly conducted and the PK information of vildagliptin was collected. PBPK modeling software, PK-Sim^®^, was then used to build and assess the IV, oral, and drug-specific models. Next, the average fold error, visual predictive checks, and predicted/observed ratios were used for the assessment of the robustness of the model for all the essential PK parameters. This evaluation demonstrated that all PK parameters were within an acceptable limit of error, i.e., 2 fold. Also to display the influence of CKD on the total and unbound AUC (the area under the plasma concentration–time curve) and to make modifications in dose, the analysis results of the model on this aspect were further examined. This PBPK model has successfully depicted the variations of PK of vildagliptin in healthy subjects and patients with CKD, which can be useful for medical practitioners in dosage optimization in renal disease patients.

## 1. Introduction

Physiologically based pharmacokinetic (PBPK) models are highly effective tools for pharmaceutical fields of drug discovery and development [1]. These dosimetry models are highly adjustable, and therefore, they can be used for simulating a variety of exposure situations, even with a limited set of data [2]. PBPK models take into account the anatomical composition of living systems where organs and tissues are represented by separate compartments that are interconnected via mass transport, resulting in a highly complex network [3]. Through the combination of both physiological and physicochemical data, it has become a valuable tool in the development of personalized medicine [4]. These models are constructed from a set of differential equations and give a quantitative mechanistic structure to describe the pharmacokinetics (PK) of drugs [5]. They incorporate drug-specific parameters combined with population-related system parameters in the model to predict the drug PK [5]. 

PBPK models are superior in their advantage over the previous empirical models as they emphasize considerably the real physiology of the organism [6] and characterize the PK of medicines using equations that were so difficult to solve mathematically in earlier times [7]. In addition to this, it is also possible to forecast the outcomes beyond a single dosage and time course range because of the extensive data set required to develop the models. This makes them valuable for simulating relationships between administered doses and concentrations in target tissues, thereby bridging the gap between empirical models and realistic physiological understanding [8].

Teorell’s model is among the pioneering PBPK models, showcasing a fundamental five-compartment structure elucidating the circulatory system, a reservoir for drugs, fluid volume, elimination through the kidneys, and tissue inactivation [9]. Nevertheless, in recent decades, numerous PBPK models have been successfully formulated for diverse drug classes [7,10,11,12,13,14,15,16]. In chronic diseases, frequent pathophysiological alterations can lead to unfavorable changes in drug PK that can be integrated into established PBPK models to modify doses in diseased populations [11].

One of the chronic diseases is type 2 diabetes mellitus (T2DM), in which vildagliptin is administered orally as a treatment option. It is a specific inhibitor of dipeptidyl peptidase-4 (DPP-4) and is used alone or in conjunction with other medications for diabetes [17]. Vildagliptin demonstrates rapid absorption, achieving peak plasma concentrations within 1 to 2 h, and displays dose-proportional PK [18]. It undergoes primary metabolism through hydrolysis in the liver, and its predominant excretion takes place through urine (85%) and feces (15%), depicting a renal clearance (CL_R_) of 13 L/h [19,20]. In the case of chronic kidney disease (CKD), the CL_R_ of vildagliptin is decreased leading to increased exposure to the drug [21].

CKD is identified by a decline in kidney function, indicated by a glomerular filtration rate (GFR) lower than 60 mL/min per 1.73 m^2^. This condition endures for a minimum of three months, irrespective of its root cause [22]. It causes abnormalities in urine, structural anomalies, or compromised renal excretory function, signaling a reduction in the number of functional nephrons [23]. In individuals with CKD, various pathophysiological alterations affect plasma protein levels (especially albumin) and hematocrit [24,25]. These changes can potentially affect the PK of vildagliptin and exacerbate its associated side effects. The most common side effects are nausea, peripheral edema, weight gain, headache, dizziness, upper respiratory infection, back pain, and diarrhea [26]. As in CKD, the exposure of vildagliptin is increased, thereby this can potentially increase the risk and severity of its associated side effects. Integrating these adjustments into the existing drug-disease model, provides a pathway for optimizing vildagliptin dosages, specifically for individuals with CKD.

There are a limited number of available publications concerning PK models for vildagliptin [27,28,29,30,31,32]. Previously there are two reports for PBPK models of DPP-4 inhibitors, among which one is focused on only modified release formulation of vildagliptin [32] and the other on other members of this class (excluding vildagliptin) [33]. Clinical studies are an important source to gather information regarding drug PK but these are also limited by certain factors, e.g., the number of subjects, demographics, or ethical considerations in silico models are great tools to assess and predict drug PK in disease populations (e.g., CKD) with scalability to provide dosage modification. This research aims to systematically create and assess a PBPK model, capable of forecasting vildagliptin PK in healthy individuals and those with CKD, with the intent of suggesting appropriate dosage modifications.

## 2. Results

**Figure 1:**
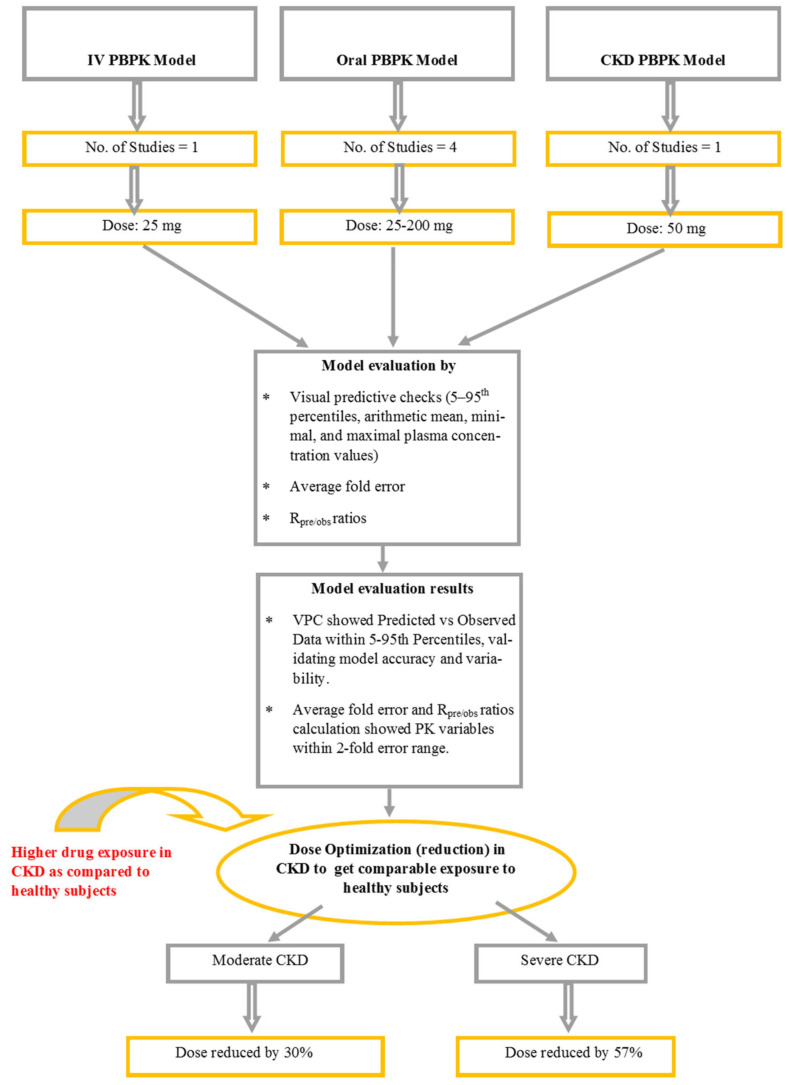
Algorithm outlining the strategic approach to achieve results. IV: intravenous, PBPK: Physiologically based pharmacokinetic model, CKD: Chronic Kidney disease, R_pre/obs_ ratio: Predicted value/observed value.

The strategic approach used to obtain the results is illustrated in Figure 1.

### 2.1. Evaluation of the PBPK Model in Healthy Subjects

**Figure 2:**
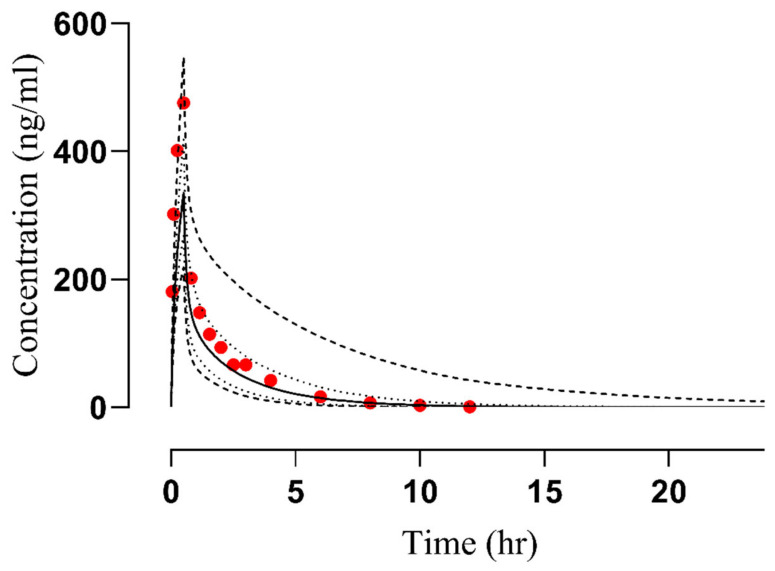
Comparison of observed and predicted plasma concentration vs. time profiles after the intravenous administration of 25 mg dose of vildagliptin [30], (…): Values of reported data, (—): Values of simulated data, (- - -): highest and lowest values, (. . .): 5th and 95th percentile.

**Figure 3:**
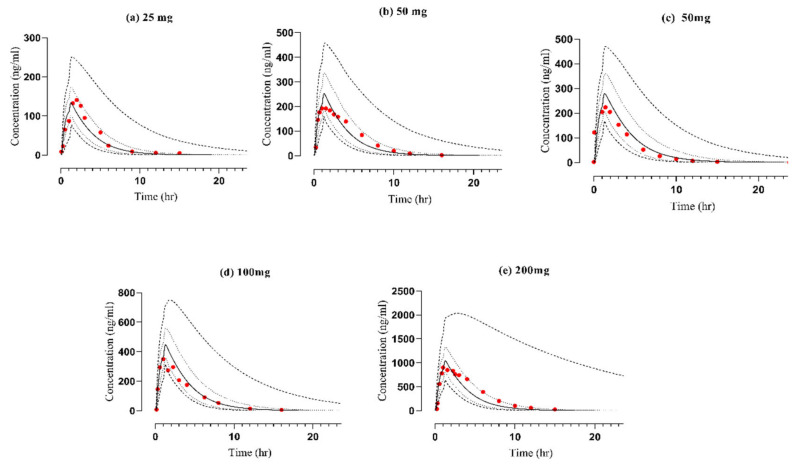
Comparison of observed and predicted plasma concentration vs time profiles of vildagliptin at oral doses (in mg) of (**a**) 25 [35], (**b**) 50 [30], (**c**) 50 [36], (**d**) 100 [37], and (**e**) 200 [38], respectively. (…): Values of reported data, (—): Values of simulated data, (- - -): highest and lowest values, (. . .): 5th and 95th percentile.

**Figure 4:**
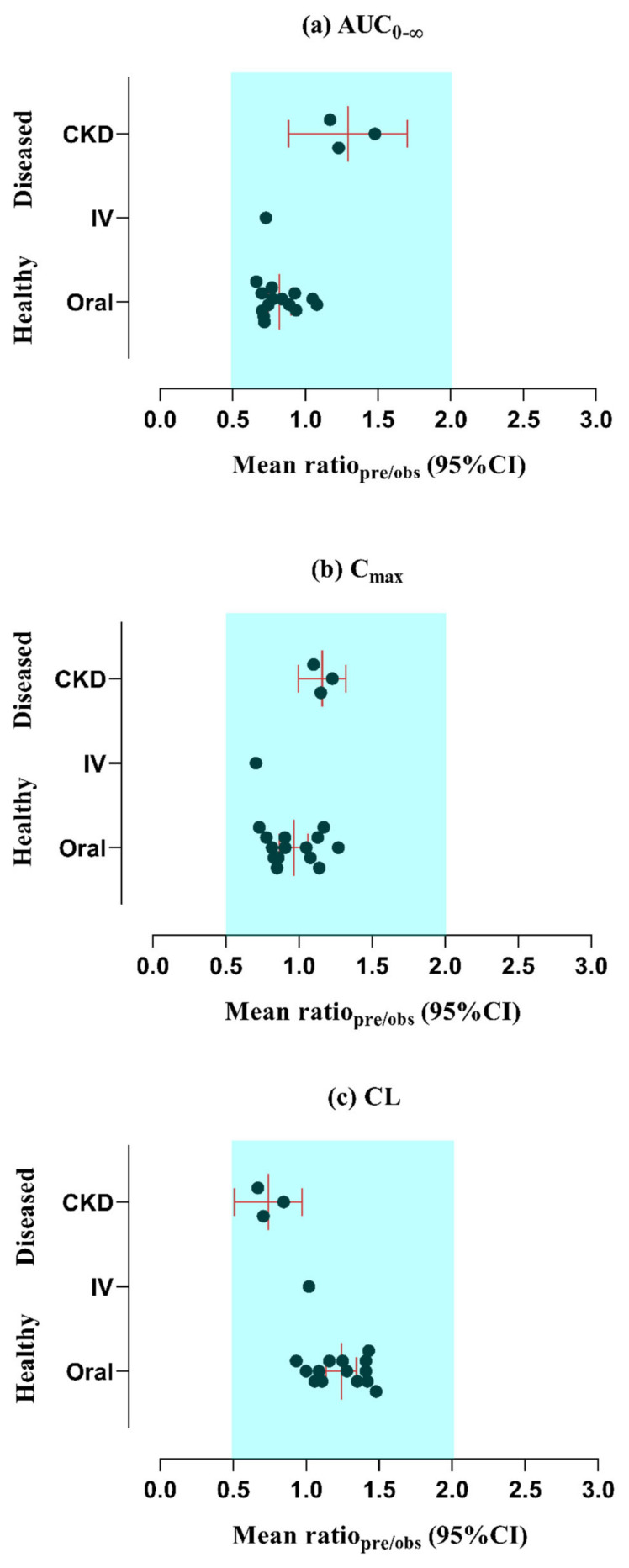
Comparison of mean R_pre/obs_ ratios for (**a**) peak plasma concentration (C_max_) (**b**) area under the curve from time 0 to infinity (AUC_0–∞_), and (**c**) clearance (CL) between healthy and diseased (CKD) patients. The red line shows the mean along with the 95% confidence interval (C.I).

Following the administration of vildagliptin via both intravenous (25 mg) and oral (25−200 mg) routes, the observed results closely matched the predicted data within the 5−95th percentile range that was evident in the concentration–time profiles (refer to Figure 2 and Figure 3 and Supplementary Figures S1 and S2). Additionally, an assessment of the developed vildagliptin model was confirmed by the average fold error (AFE) value which indicates how much a model’s predictions typically deviate from the actual values [34], revealing a peak plasma concentration (C_max_) of 1.035 following oral administration. Furthermore, the PK variables, including clearance (CL) and the area beneath the curve from time 0 to infinity (AUC_0–∞_), were similar, falling inside the optimal error range of 2-fold (refer to Table 1 and Figure 4). The calculations of AUC_0–∞_, C_max_, and CL were conducted using mean predicted/observed (R_pre/obs_) ratios, as detailed in Table 2. 

The model predictions were segregated on the basis of gender to see any differences in the predictions and it was seen that gender had no significant effect on vildagliptin PK (Supplementary Figure S3). The included studies are focused on various population ethnicities, i.e., Hispanic, Chinese, Black, or Caucasian. A comparison of observed and predicted plasma concentration vs time profiles of vildagliptin in different population ethnicities is given in Supplementary Figure S4.

### 2.2. Assessment of the PBPK Model in CKD Patients

**Figure 5:**
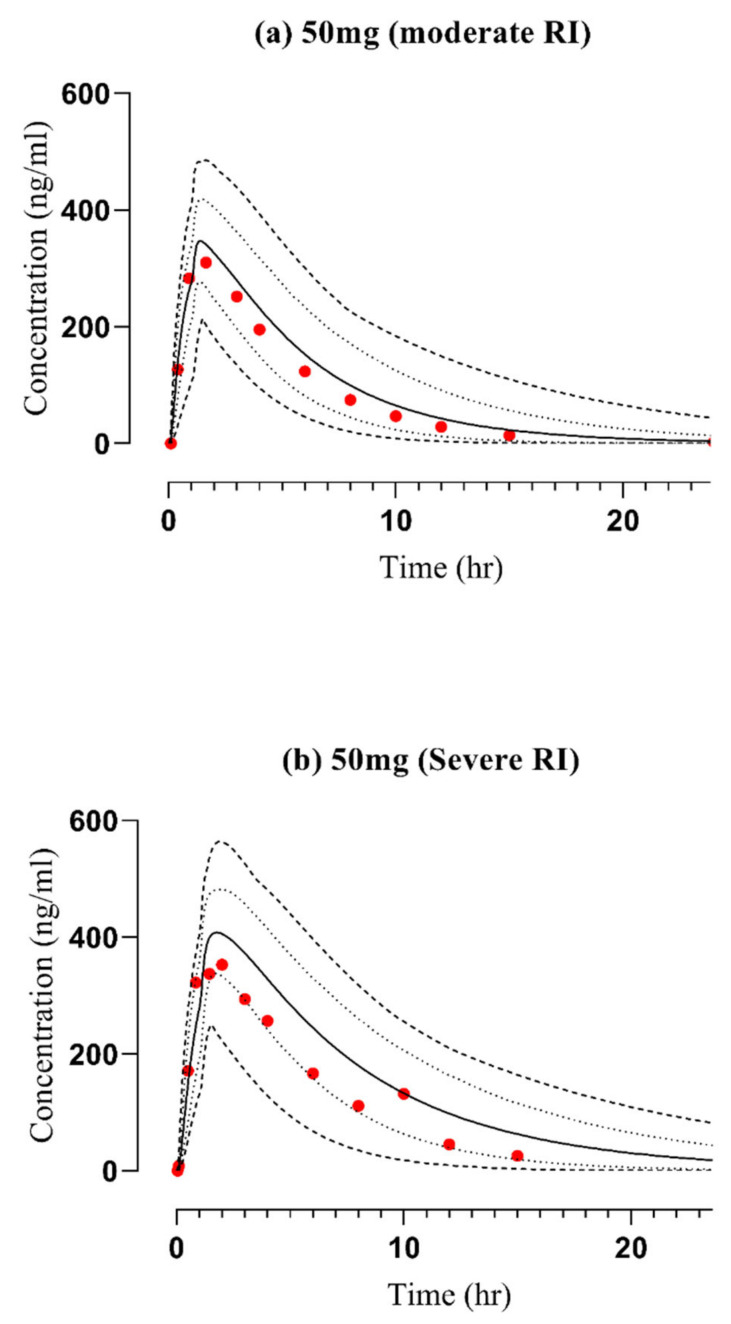
Observed and predicted concentration vs time profiles after administrating vildagliptin orally in CKD patients at doses (in mg) of (**a**) 50 mg [36] (Moderate RI) and (**b**) 50 mg [36] (Severe RI), RI: renal impairment. (…): Values of reported data, (—): Values of simulated data, (- - -): highest and lowest values, (. . .): 5th and 95th percentile.

For subjects with CKD, the observed data demonstrated concordance with the simulated systemic concentration–time profiles of vildagliptin. This result was evident when considering both the arithmetic mean and the 5−95th percentile, as depicted in Figure 5. The differences in half-life among healthy and diseased subjects are presented in Supplementary Table S1. AFE values (presented in Table 2) and mean R_pre/obs_ ratios were calculated for essential PK variables in order to confirm the reliability of these results. Notably, as shown in Figure 4 and explained in Table 1, all of these ratios were determined to be in the 2-fold acceptable error range. 

### 2.3. Vildagliptin Dose Modification in CKD Patients

**Figure 6:**
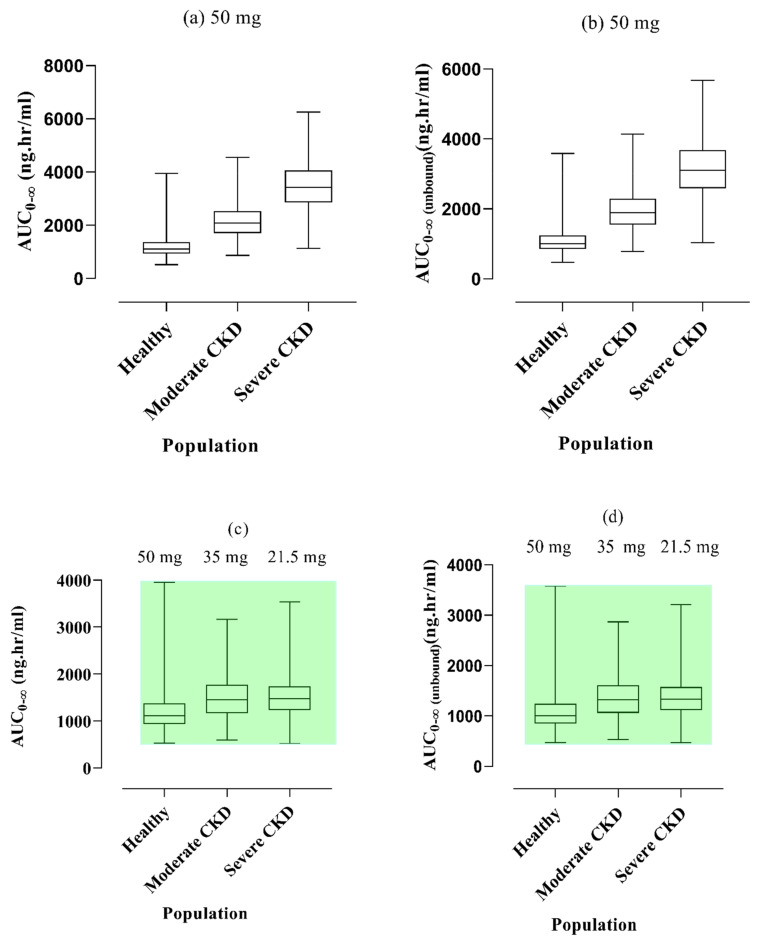
Box plots showing, the simulated AUC_0–∞_ and AUC_0–∞ (unbound)_ with 5–95th percentiles after orally giving 50 mg dose of vildagliptin in both the healthy and renal failure populations (**a**,**b**). Dosage reduction for moderate and severe renal failure is suggested in (**c**,**d**) for comparison with healthy exposure. AUC_0–∞ (unbound)_: area under the curve from time 0 to infinity unbound, AUC_0–∞_: area under the curve from time 0 to infinity bound, CKD: Chronic kidney disease.

When the same dosage of vildagliptin was given orally to people with CKD and those in good health, the CKD population exhibited higher values for AUC_0–∞_ and AUC_0–∞ (unbound)_. To achieve comparable exposure to vildagliptin in both healthy participants and CKD patients, a step-by-step process of dosage modification was conducted through various simulations, incorporating gradual tapering. The exposure levels became equivalent when the dose was reduced to 57% of the initial 50 mg dose, as depicted in box plots for severe renal impairment. Conversely, in cases of moderate kidney impairment, the dosage was decreased to around 30%. Notably, no notable differences were noted in the optimization of doses, specifically concerning AUC_0–∞_ and AUC_0–∞ (unbound)_ as shown in Figure 6.

## 3. Discussion

An accurate PBPK model for vildagliptin’s PK has been presented in this study that was established through a comprehensive and structured approach, allowing the anticipation of PK in healthy, and CKD populations following both IV and oral administration of vildagliptin. The initial phase involved the proficient creation and analysis of the PBPK model in healthy subjects, aligning with insights from previously published investigations [7,10,11,12,13,14,15,16]. 

The PBPK model was developed and evaluated using the PK-Sim^®^ software, which has given researchers a thorough understanding of how vildagliptin is disposed of. The real and predicted values matched well, with the average C_max_ after oral administration to healthy volunteers recorded at 486 ng/mL in actual data and 446 ng/mL in the simulation. The AFE value for CL after oral vildagliptin administration was 1.238, falling within the 2-fold range of error, demonstrating that the model has precisely depicted the ADME (absorption, distribution, metabolism, and elimination) through accurate input parameters. Furthermore, the mean R_pre/obs_ ratio for AUC_0–∞_ was 0.82 in the healthy population which showed the model’s accurate predictive capabilities regarding vildagliptin PK.

Given that vildagliptin is primarily eliminated through the kidneys [39], its PK may be influenced in the presence of renal dysfunction. In individuals with CKD, variability in various parameters, including kidney volume, gastric emptying time, small intestinal transit time, hematocrit, and albumin, is commonly observed, as highlighted in earlier studies [24,25]. In CKD cases, both simulated and reported values of vildagliptin AUC_0–∞_ indicated an increase in CKD patients, escalating to 2277.33 ng.h/mL from 1711.33 ng. h/mL after oral administration, aligning with the findings of the previous study [25]. These observations suggest that CKD significantly influences pathophysiological changes by decreasing CL and elevating plasma concentration levels. In analyzing CKD profiles, a comparison was made between two parameters, namely AUC_0–∞ (unbound)_ and AUC_0–∞_, revealing a need for a 30% dose reduction in severe cases and 57% in moderate cases, respectively. These modifications are very close to the literature values that depicted a 50% decrease in vildagliptin dose in moderate and severe CKD populations as compared to healthy populations [40]. These projections for vildagliptin doses could potentially assist patients with CKD in mitigating the risk of exacerbating their condition.

## 4. Limitations

One notable limitation in our research paper pertains to the inadequacy of available data for patients with mild renal impairment. This scarcity of relevant information has a direct impact on the overall robustness of this PBPK model, thereby warranting caution and acknowledging a potential limitation in the generalizability of our findings to this particular population subset. As the optimization was conducted for the Log P value to 1.55 log units to improve the accuracy of the model, this adjustment highlights one other limitation of the PBPK model. Thirdly, the accuracy of the model could be further compromised due to not testing the IV dosage form in CKD patients, and that would affect the generalizability of the results. Another limitation of our study is that each of the five studies focused on a single ethnic group, e.g., Hispanic, Chinese, Black, or Caucasian, which may affect the generalizability of our findings due to potential variations in dipeptidyl peptidase-4 enzyme parameters across these populations.

## 5. Methodology

### 5.1. Screening of Pharmacokinetic Parameters

A thorough search was conducted across multiple databases, including Google Scholar, PubMed, Science Direct, and EBSCO, to gather articles related to the PK of vildagliptin. The focus was on its administration by IV and oral routes, with a specific emphasis on systemic concentration–time profiles in healthy and diseased populations. Regarding healthy individuals, 4 studies were included, comprising 1 profile related to IV infusion and 15 profiles related to oral administration. Additionally, a PK study with three profiles, showing drug concentration vs. time data in individuals with CKD, was included. Subsequently, version 2.26 of Get Data Graph Digitizer software was utilized to extract numerical data from graphs in articles that fulfilled the inclusion criteria. During the development phase of the model, one-third (2 studies) of the studies were utilized, while the remaining two-thirds (3 studies) were dedicated to the evaluation process. The specific attributes of all the included research articles are outlined below in Table 3.

### 5.2. Software System for Modeling

For the construction and assessment of the PBPK model for vildagliptin, the simulation software PK-Sim^®^, notably version 11.2-build 142, crafted by Bayer Technology Services, in Wuppertal, Germany, was utilized.

### 5.3. Building Blocks Creation

PK-Sim^®^ software features an intuitive graphical user interface, integrating diverse building blocks to facilitate seamless operation and modeling. The model configuration for PK parameters of vildagliptin under various conditions, including both healthy and diseased states, along with drug-specific parameters, was established using values extracted from the articles, as outlined in Table 4.

### 5.4. Model Development Strategy

**Figure 7:**
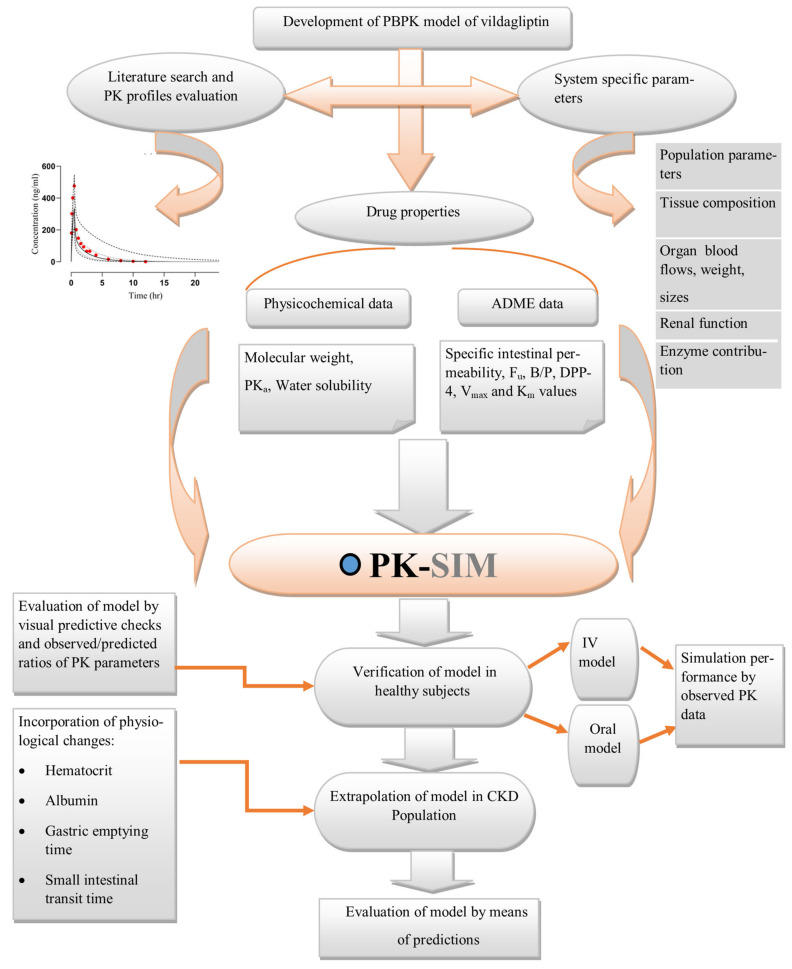
PBPK model development strategy. ADME: absorption, distribution, metabolism, and elimination, pKa: dissociation rate constant, f_u_: unbound fraction, B:P: blood to plasma ratio, V_max_: maximum velocity of reaction, K_m_: concentration of substrate at half of the maximum velocity, DPP-4: dipeptidyl peptidase-4, PK: Pharmacokinetics.

The construction of the PBPK model for vildagliptin commenced with an extensive literature search to identify PK data. Then, PK profiles, system-related variables, and drug parameters were incorporated into the PK-Sim^®^ to validate the model in a healthy population. This process included the development of both IV and oral models, employing established model-building techniques derived from prior research [7,10,11,12,13,14,15]. Initially, the IV model was constructed, and then without modifying parameters, the oral model was developed to circumvent the complexities associated with absorption parameters such as specifically intestinal permeability. To further extend the model’s applicability to diseased populations, specifically those with CKD, various pathophysiological changes were then incorporated into the model. The graphical depiction of this modeling approach is presented in Figure 7.

### 5.5. Structure of Model

Vildagliptin is characterized by the molecular formula C_17_H_25_N_3_O_2_ with a fraction unbound (f_u_) of 90.7% [37,42,45]. All other parameters used for model development can be found in Table 4.

### 5.6. Configuration of the PBPK Model in the Diseased Population (CKD)

In the moderate and severe CKD profiles, the eGFR was included in the model as 48 mL/min/1.73 m^2^ and 29 mL/min/1.73 m^2^, respectively. To further refine the model, the changes in various pathophysiological parameters were incorporated into the virtual populations generated in the PK-Sim program. To incorporate the plasma protein binding changes in CKD, a plasma protein scaling factor of 0.93 and 0.83, respectively, was incorporated into the moderate and severe CKD populations. The area under the plasma concentration–time curve from time 0 to infinity AUC_(0–∞)_, and AUC_(0–∞)unbound_ were compared across three groups of CKD patients (healthy, moderate, and severe) following the assessment of the model by comparing it with observed profiles. Additionally, box-whisker plots were used to provide visual depiction and recommendations for vildagliptin dosage.

### 5.7. Verification of the Model

For each PK profile, a simulated cohort of 1000 individuals was created, incorporating key attributes from clinical PK studies. These attributes encompassed variables such as gender distribution, age, administered dosage, body weight, and route of administration. Subsequently, visual predictive checks (VPC) were utilized to evaluate the developed vildagliptin model. It is a method used to validate PK models by visually comparing simulated data against observed data, typically plotted over time or another independent variable. It assesses how well the model predicts the variability and central tendency of real-world data [47]. This involved overlaying datasets from already published PK profiles onto expected data, including the 5–95th percentiles, arithmetic mean, minimal, and maximal concentration values. Additionally, non-compartmental analysis was conducted using the PK Solver add-in for Microsoft Excel 2013. This allowed for the prediction of PK parameters such as C_max_, AUC_0–∞_, and CL for both reported and predicted data [48]. In studies involving healthy subjects, R_pre/obs_ ratios for each of the PK variables (AUC_0–∞_, C_max_, and CL) were calculated using equation 1 (presented below), with a 95% C.I. However, data was displayed as mean and range in studies involving diseased (CKD) subjects because there were only two studies available. The previously developed PBPK models predict that these ratios should be within a 2-fold error range [7,10,11,12,13,14,15,16]. Additionally, using Equations (1)–(3), mean R_pre/obs_, AFE, and fold error were calculated for model accuracy assessment.

(1) $$R=\frac{Predicted\;value\;of\;PK\;parameter}{observed\;value\;of\;PK\;parameter},$$

(2) $$\mathrm{Fold}-\mathrm{error}=\frac{Predicted\;values\;of\;parameter}{observed\;values\;of\;parameter},$$

(3) $$\mathrm{AFE}=10^{\frac{\sum \log\left(fold\;error\right)}{N}}.$$

## 6. Conclusions

The PBPK model has accurately forecasted the ADME of vildagliptin, demonstrating its efficacy not only in healthy individuals but also in those with CKD. By incorporating a range of pathophysiological alterations linked to CKD, the model has been augmented to anticipate outcomes more reliably, providing clinicians with crucial guidance in dose optimization for patients with compromised kidney function.

## Figures and Tables

**Table 1:** R_pre/obs_ ratios for PK parameters of Vildagliptin.

Dose (mg)	C_max_ (ng/mL)			AUC_0–∞_ (ng·h/mL)			CL/F (L/h)			Ref.
	OV	PV	R Ratio	OV	PV	R Ratio	OV	PV	R Ratio	
Oral administration in Healthy individuals										
25 ^a^	140	127	0.90	566	507	0.89	44	49	1.11	[35]
25 ^b^	138	125	0.90	541	507	0.93	46	49	1.06	[35]
25	112	127	1.13	475	499	1.05	50	50	1.00	[38]
50 ^a^	294	254	0.86	1269	980	0.77	39	50	1.28	[35]
50 ^b^	339	250	0.73	1241	962	0.77	40	50	1.25	[35]
50	212	242	1.14	1164	989	0.84	42.9	50	1.16	[38]
50	191	243	1.27	1097	1019	0.92	45	49	1.08	[30]
100 ^a^	654	509	0.77	2799	2010	0.71	35	49.7	1.42	[35]
100 ^b^	610	509	0.83	2667	1987	0.74	37	50	1.35	[35]
100	446	484	1.08	2670	1871	0.70	37	53	1.43	[38]
100	350	410	1.17	1630	1767	1.08	60	56	0.93	[37]
200 ^a^	1247	1019	0.81	5776	4073	0.70	34.6	49	1.41	[35]
200 ^b^	1173	1001	0.85	5760	4125	0.71	34	48	1.41	[35]
200	902	953	1.05	5314	3525	0.66	37.6	56	1.48	[38]
IV administration in Healthy individuals										
25	476	337	0.70	660	479	0.73	51	52.1	1.02	[30]
Oral administration in the CKD population										
50	224	277	1.23	976	1204	1.23	51	40	0.78	[36]
50 (Mod)	310	342	1.10	1792	2107	1.17	27.8	23.6	0.84	[36]
50 (severe)	353	406	1.15	2366	3519	1.48	21	14.2	0.67	[36]

AUC_0–∞_: area under the concentration–time curve from time 0 to infinity, C_max_: maximum plasma concentration, CL/F: total body clearance, Ref.: reference, OV: Observed value, PV: Predicted Value, R ratio: Predicted value/Observed value, CKD: chronic kidney disease, Mod: moderate CKD, IV: intravenous, ^a^ Drug administration at Day 1, ^b^ Drug administration at Day 13.

**Table 2:** Calculating the AFE values for each PK variable in participants with CKD and those in good health.

PK Parameters	AFE
Oral healthy	
C_max_ (ng/mL)	1.03
AUC_0–∞_ (ng·h/mL)	1.21
CL (L/h)	1.23
Renal failure	
C_max_ (ng/mL)	1.15
AUC_0–∞_ (ng·h/mL)	1.28
CL (L/h)	1.30

AUC_0–∞_: area under the concentration–time curve from time 0 to infinity, C_max_: maximum plasma concentration, CL: total body clearance, PK: pharmacokinetics, AFE: average fold error, CKD: Chronic kidney disease.

**Table 3:** Comprehensive demographic and dosage overview of included studies.

Sr. No.	Reference	Gender	Female Proportion (%)	Age (Years)	Population	Route	Dose (mg)	Study Size	Weight (kg)
1	[30]	M and F	45	18–45	HT	PO	50	11	68.1 ± 7.1 ^c^
						IV	25		
2	[35]	M and F	48	18–45	HT	PO	25, 50, 100, or 200	60	58.6 ± 5.6 ^c^
3	[38]	M and F	65	18–45	HT	PO	25, 50, 100, 200	20	70.9 ± 8.6 ^c^
4	[37]	M	0	18–45	HT	PO	100	4	77–93 ^a^
5	[36]	M and F	Mild: 56	18–85	CKD	PO	50	96	Mild: 63.1 ^b^
			Moderate: 62						Moderate: 62.4 ^b^
			Severe: 61						Severe: 63.7 ^b^

^a^ Range, ^b^ Average, ^c^ Data presented with Standard deviation, CKD: chronic kidney disease, HT: healthy, M: male, Ref: reference, PO: per oral, IV: intravenous, F: female.

**Table 4:** Model development input parameters for vildagliptin.

Parameters for Model	Values of Literature	Input Values	Reference
Physical and Chemical Properties			
Plasma protein binding	Albumin	Albumin	[41]
Molecular weight (g/mol)	303.4	303.4	[42]
Water solubility (mg/mL) at pH 7	60	60	[43]
pKa (base)	9.7	9.7	[37]
Log P	1.12	1.55 *	[44]
Absorption			
Specific intestinal permeability (cm/s)	5 × 10^−4^	5 × 10^−4^	[32]
Formulation method	Weibull		
Dissolution Time (50% dissolved) (minutes)	50		Optimized
Distribution			
Fraction unbound (f_u_)	90.7%	90.7%	[45]
Partition Coefficient Model	Poulin Theil		
Cellular Permeability Model	PK Sim Standard		
Metabolism			
Vildagliptin K_m_ (uM)	190	190	[46]
Vildagliptin V_max_ (nmol/L/s)	23.31	23.31	[46]
Excretion			
Renal clearance (L/h)	13	13 **	[30]
	Healthy	CKD	
eGFR	>60 mL/min	Moderate: 48 mL/min Severe: 29 mL/min	[24]
Gastric Emptying Time	15 min	Moderate: 20.63 min Severe: 39 min	[24]
GIT Transit Time	2.1 h	Moderate: 2.94 h Severe: 4.12 h	[24]
Hematocrit	0.47	Moderate: 0.42 Severe: 0.37	[24]

pKa: dissociation rate constant, V_max_: maximal reaction velocity, K_m_: concentration of substrate at half of the maximum velocity, f_u_: fraction unbound, log P: Lipophilicity, min: minutes, h: hour, CKD: chronic kidney disease. * This value underwent manual optimization guided by visual predictive checks and the R_pre/obs_ ratios. ** Converted to L/h/kg, the model utilizes a value of 0.18.

## Data Availability

All the data generated during the research is reported in the manuscript.

## Supplementary Materials

The following supporting information can be downloaded at: https://www.mdpi.com/article/10.3390/ph17070924/s1, Figure S1: Comparison of observed and predicted plasma concentration vs. time profiles of vildagliptin at different oral doses. Figure S2: Comparison of observed and predicted plasma concentration vs. time profiles of vildagliptin at different dosage levels. Figure S3: Comparison of observed and predicted plasma concentration vs. time profiles of vildagliptin in males and females. Figure S4: Comparison of observed and predicted plasma concentration vs. time profiles of vildagliptin in different ethnic populations. Table S1: Vildagliptin half-life (t1/2) in healthy and diseased populations. References [30,35,36,37,38] are cited in the Supplementary Materials.
