# Supplementary material for: A Comprehensive Physiologically Based Pharmacokinetic Model for Predicting Vildagliptin Pharmacokinetics: Insights into Dosing in Renal Impairment
Source: Pharmaceuticals (Basel). 2024 Jul 10;17(7):924. doi: 10.3390/ph17070924 (PMC11280059; doi:10.3390/ph17070924)
Supplement: Supplementary file 1 [file pharmaceuticals-17-00924-s001.zip › pharmaceuticals-3050040-supplementary.pdf]

A Comprehensive Physiologically Based Pharmacokinetic Model for Predicting Vildagliptin Pharmacokinetics: Insights into Dosing in Renal Impairment

Mahnoor Pasha [1], Ammara Zamir [1], Muhammad Fawad Rasool [1*], Hamid Saeed [2], Tanveer Ahmad [3], Nawaf Shalih Alqahtani [4], Lamya Saif Alqahtani [5] and Faleh Alqahtani [6*]

[1]Department of Pharmacy Practice, Faculty of Pharmacy, Bahauddin Zakariya University, 60800, Multan, Pakistan; mahnoorpashaoc15@gmail.com (M.P); ammarazamir20@gmail.com (A.Z); fawadrasool@bzu.edu.pk (M.F.R)
[2] Section of Pharmaceutics, University College of Pharmacy, Allama Iqbal Campus, University of the Punjab, 54000, Lahore, Pakistan; hamid.pharmacy@pu.edu.pk (H.S)
[3] Institute for Advanced Biosciences (IAB), CNRS UMR5309, INSERM U1209, Grenoble Alpes University, La Tronche, 38700, France; tanveer.ahmad@univ-grenoble-alpes.fr (T.A)
[4]King Abdulaziz Medical City- Riyadh Region Ministry of National Guard, Health Affairs; Alkahtanina3@mngha.med.sa (N.S.A)
[5]Department of Cardiology, Prince Sultan Cardiac Center, Riyadh, 11625, Saudi Arabia; lalqahtani@pscc.med.sa (L.S.A)
[6]Department of Pharmacology and Toxicology, College of Pharmacy, King Saud University, Riyadh, 11451, Saudi Arabia; afaleh@ksu.edu.sa. (F.A)
* Correspondence: fawadrasool@bzu.edu.pk (M.F.R), afaleh@ksu.edu.sa (F.A)

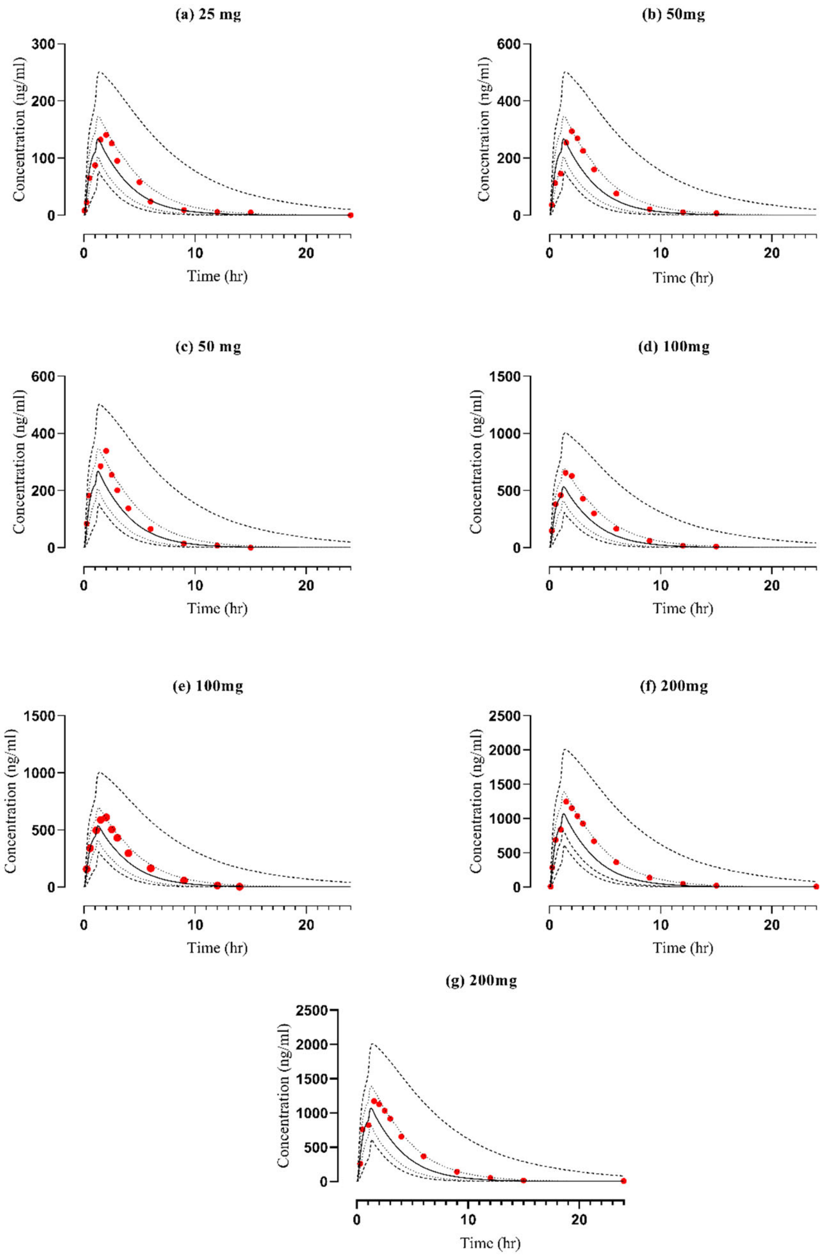

Figure S1: Comparison of observed and predicted plasma concentration vs time profiles of vildagliptin at different oral doses. (Dose in mg) (a) 25[1] , (b) 50[1], (c) 50[1], (d) 100[1] and **(e)** 100[1], (f) 200[1], and (g) 200[1] respectively. (…): Values of reported data, (—): Values of simulated data, (- - -): highest and lowest values, (...): 5th and 95th percentile.

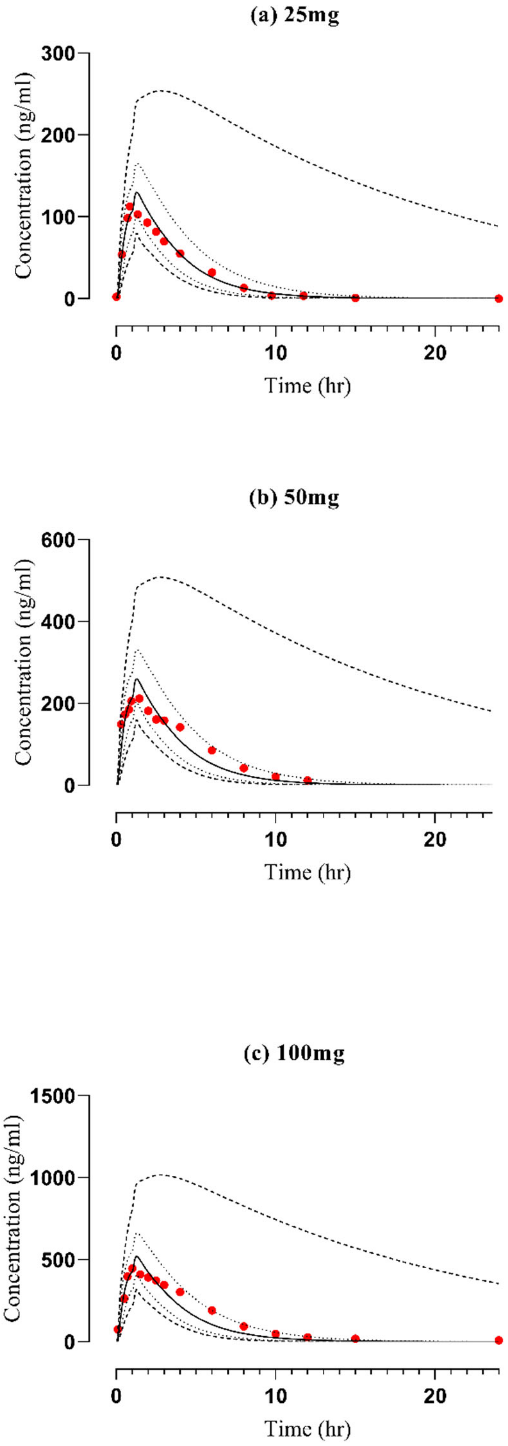

Figure S2: Comparison of observed and predicted plasma concentration vs time profiles of vildagliptin at different dosage levels. (Dose in mg) (a) 25[2] , (b) 50[2], and (c) 100[2] respectively. (…): Values of reported data, (—): Values of simulated data, (- - -): highest and lowest values, (...): 5th and 95th percentile

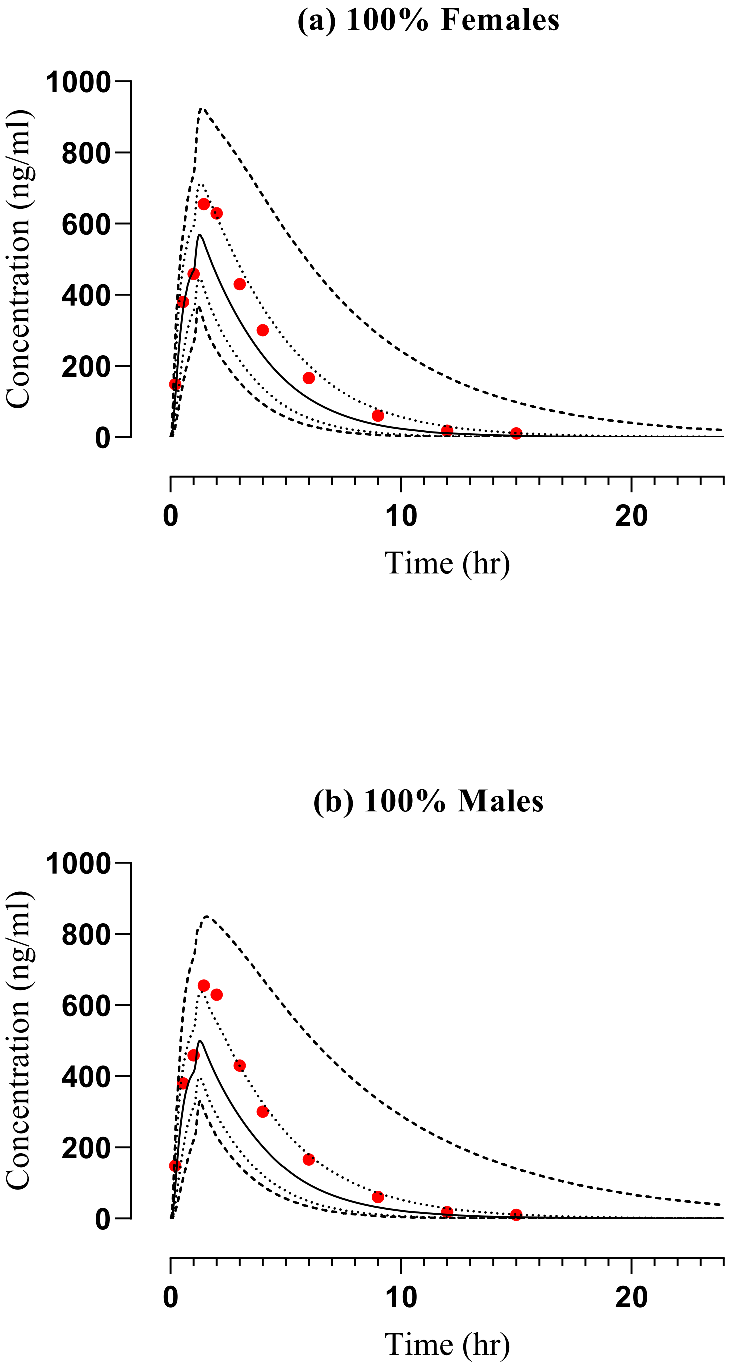

Figure S3: Comparison of observed and predicted plasma concentration vs time profiles of vildagliptin in males and females. Oral dose of 100 mg[1] showing that gender imbalance has no effect on simulations**.** (…): Values of reported data, (—): Values of simulated data, (- - -): highest and lowest values, (...): 5th and 95th percentile

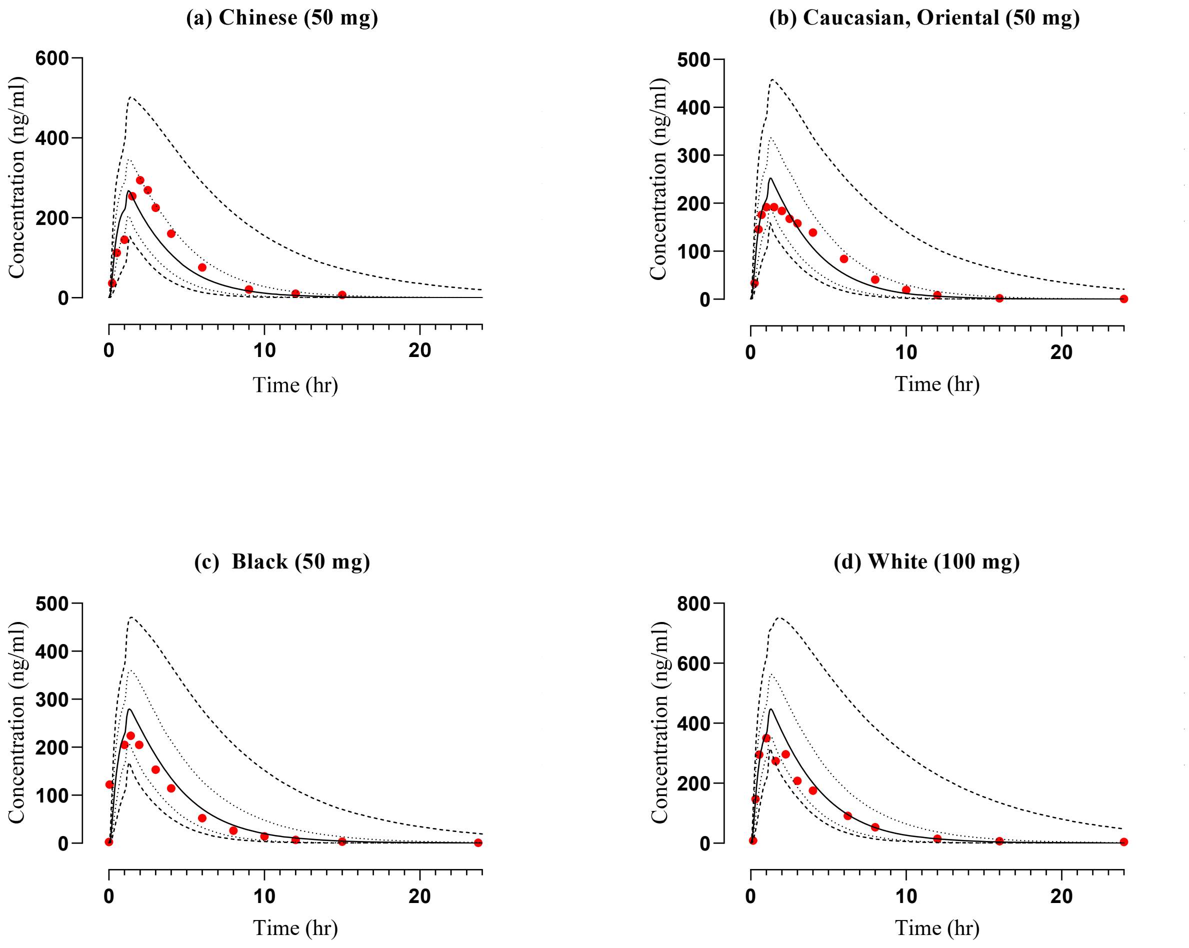

Figure S4: Comparison of observed and predicted plasma concentration vs time profiles of vildagliptin in different ethnic populations (…): Values of reported data, (—): Values of simulated data, (- - -): highest and lowest values, (...): 5th and 95th percentile

Table S1: Vildagliptin half-life ($t_{1/2}$) in healthy and diseased population

Dose (mg)	Half Life ($t_{1/2}$) from predicted data	Reference
**Oral administration in Healthy individuals**		
25[a]	2.07	[1]
25[b]	2.06	[1]
25	2.13	[2]
50[a]	1.98	[1]
50[b]	1.92	[1]
50	1.93	[2]
50	1.95	[3]
100[a]	2.06	[1]
100[b]	2.01	[1]
100	2.1	[2]
100	2.08	[4]
200[a]	2.06	[1]
200[b]	2.11	[1]
200	2.13	[2]
**IV administration in Healthy individuals**		
25	2.06	[3]
**Oral administration in the CKD population**		
50	1.99	[5]
50 (Mod)	3.30	[5]
50(severe)	4.88	[5]

References

1. He, Y., et al. Pharmacokinetics and pharmacodynamics of vildagliptin in healthy Chinese volunteers. in CLINICAL PHARMACOLOGY & THERAPEUTICS. 2008. NATURE PUBLISHING GROUP 75 VARICK STREET, 9TH FLOOR, NEW YORK, NY 10013-1917 USA.

2. Sunkara, G., et al., Dose proportionality and the effect of food on vildagliptin, a novel dipeptidyl peptidase IV inhibitor, in healthy volunteers. J Clin Pharmacol, 2007. 47(9): p. 1152-8.

3. He, Y.-L., et al., The absolute oral bioavailability and population-based pharmacokinetic modelling of a novel dipeptidylpeptidase-IV inhibitor, vildagliptin, in healthy volunteers. Clinical pharmacokinetics, 2007. 46(9): p. 787-802.

4. He, H., et al., Absorption, metabolism, and excretion of [14C] vildagliptin, a novel dipeptidyl peptidase 4 inhibitor, in humans. Drug metabolism and disposition, 2009. 37(3): p. 536-544.

5. He, Y.L., et al., Pharmacokinetics of vildagliptin in patients with varying degrees of renal impairment. Int J Clin Pharmacol Ther, 2013. 51(9): p. 693-703.
